# Perspectives for Tibetan Plateau data assimilation

**DOI:** 10.1093/nsr/nwaa014

**Published:** 2020-02-06

**Authors:** Zong-Liang Yang, Long Zhao, Yujun He, Bin Wang

**Affiliations:** 1 Department of Geological Sciences, Jackson School of Geosciences (JSG), the University of Texas at Austin, USA; 2 Key Laboratory of Regional Climate-Environment Research for Temperate East Asia (RCE-TEA), Institute of Atmospheric Physics, Chinese Academy of Sciences, China; 3 School of Geographical Sciences, Southwest University, China; 4 State Key Laboratory of Numerical Modeling for Atmospheric Sciences and Geophysical Fluid Dynamics (LASG), Institute of Atmospheric Physics, Chinese Academy of Sciences, China; 5 Department of Earth System Science, Tsinghua University, China; 6 College of Ocean Sciences, University of Chinese Academy of Sciences, China

**Figure 1.**
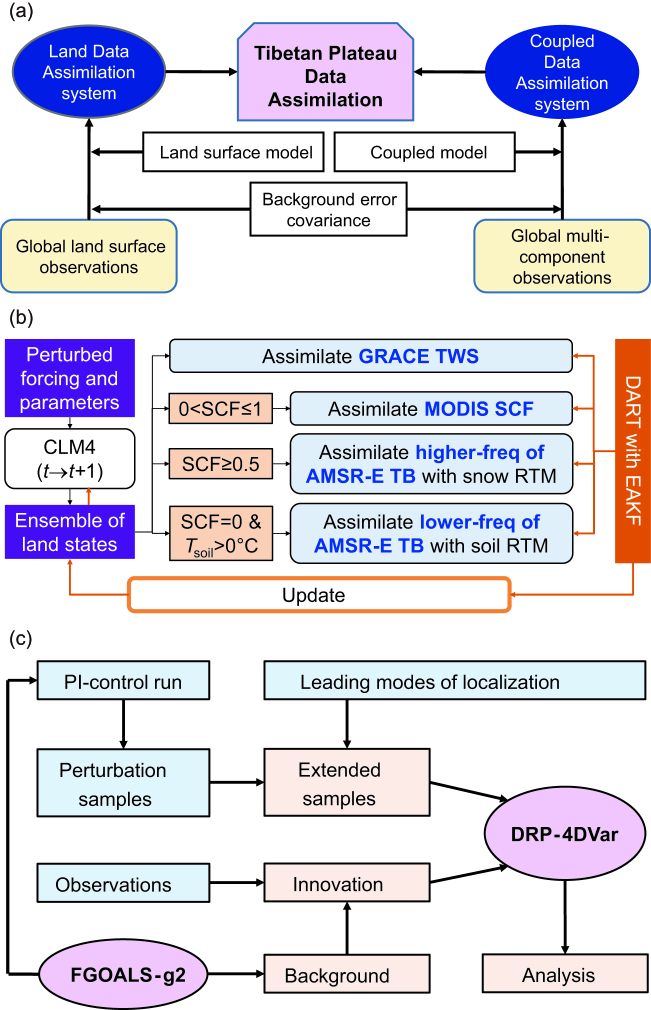
Schematics for (a) Tibetan Plateau data assimilation, (b) general design of the DART/CLM4-based multisensor land-assimilation framework and (c) DRP-4DVar-based Coupled Data Assimilation System using FGOALS-g2. Note that the black and orange arrow lines in (b) indicate the forecast and analysis steps, respectively.

Known as the Third Pole, the Tibetan Plateau (TP) exhibits profound impacts on weather and climate on regional and global scales. Due to complex terrains and harsh environments, conventional observations over the TP are sparse and their qualities are poor, which significantly hinders our understanding of the TP’s influences on societies and ecosystems. In order to produce high-quality data sets, data assimilation (DA) has been pursued, as it effectively integrates information from both model predictions and multi-source observations. A TP region-specific DA system may be limited by sparseness and poor quality of local observations. A global DA system can alleviate this limitation by propagating information into the TP using either observed spatial and temporal correlations estimated in the background-error covariance or the physical relationships included in the model [1] (Fig. 1a). Therefore, the perspectives for TP DA are mainly focused on global scales.

**Table 1.** Existing typical regional and global land data assimilation systems.

DA systems	Model operator	Variables assimilated	Assimilation algorithm	Features	Institution
NASA-GLDAS***	CLM2.0, Noah, VIC, Mosaic	MODIS snow cover	Optimal Interpolation	Multi-model; long temporal coverage	NASA GSFC
ECMWF-LDAS***	HTESSEL	ASCAT soil moisture, IMS snow cover, SMOS T_B_	Simplified EKF and Optimal Interpolation	Operational for weather forecast	ECMWF
CAREERI-LDAS*	CoLM and SiB2	Microwave T_B, satellite soil moisture, LAI	Ensemble Kalman Filter and Ensemble Kalman Smoother	First LDAS in China; Multi-model and observation operators	CAREERI, CAS
IAP-LDAS*	CLM3.0	AMSR-E T_B_	POD-based ensemble 4DVAR	Simultaneously update model parameters and land states for operational weather forecast	IAP, CAS
ITP-LDAS*	SiB2	AMSR-E T_B_	Cost-function based variational DA	Optimize time-invariant and time-variant parameters/variables separately	ITP, CAS
CMA-CLDAS**	CLM3.5 and CLM4.5, Noah-MP	Microwave T_B_, satellite soil moisture/temperature	Ensemble Kalman Filter	Multi-model and multi-variate DA	NMIC

CLM, the Community Land Model; HTESSEL, Hydrology Tiled ECMWF Scheme for Surface Exchanges over Land; CoLM, Common Land Model; SiB2, Simple Biosphere Model Version 2; T_B_, brightness temperature. Symbols for spatial coverage: *, China; **, East Asia; ***, global. ECMWF-LDAS can be accessed at https://confluence.ecmwf.int/display/LDAS/; CMA-CLDAS can be accessed at http://www.nmic.cn/.

**Figure 2.**
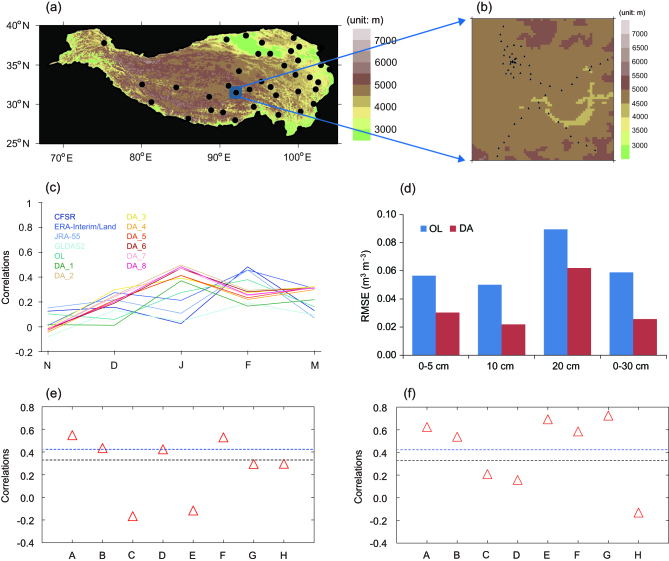
(a) Spatial distribution of China Meteorological Administration observational stations over the Tibetan Plateau area; (b) Naqu soil-moisture-monitoring network; evaluation of DART/CLM4-based multisensor DA in terms of (c) interannual seasonal snow-water equivalent (SWE) and (d) soil-moisture profile with respect to *in situ* observations; and correlation coefficients of eight decadal hindcasts of (e) precipitation and (f) surface air temperature in summer over the Tibetan Plateau against GPCC and HadCRUT4, respectively. Capital letters in the horizontal axes in (e) and (f) represent the models that conduct the hindcasts with different ensemble members. A and B, from FGOALS-g2 with 10 ensemble members and three ensemble members, respectively. C–H, from six CMIP5 models with three ensemble members: C, BCC-CSM1.1; D, CanCM4; E, CM2.1; F, HadCM3; G, MIROC5; H, MPI-ESM-LR. Note that *DA_x* (*x* = 1, 2, …, 8) in (c) stands for eight DART/CLM4 estimates by assimilating different combinations of sensors, while the blue and black dashed lines in (e) and (f) indicate the confidence levels of 99% and 95%, respectively.

Over the past decades, tremendous advances have been made at various fronts including Earth-observing satellites, high-performance computing, DA methods and state-of-the-art land-surface and atmospheric modeling. Taking advantage of efforts from existing regional and global land DA (LDA) systems (Table 1), Zhao and Yang [2] developed a prototype multisensor and multivariate LDA system based on the National Center for Atmospheric Research (NCAR) Community Land Model Version 4 (CLM4) and the Data Assimilation Research Testbed (DART) (Fig. 1b). Moderate Resolution Imaging Spectroradiometer (MODIS) snow-cover fraction (SCF), Advanced Microwave Scanning Radiometer—Earth Observing System (AMSR-E) brightness temperature (T_B_) and Gravity Recovery and Climate Experiment (GRACE) terrestrial water storage (TWS) change were assimilated either individually or in combination, and there were improvements in estimating snow mass and soil moisture over the TP (Fig. 2a–d). The potential for finding an optimal combination of satellite sensors for snow estimation with regard to specific regions and the necessity for combing various sources of satellite observations for multisensor LDA were emphasized [2].

Zhang and Yang [3] systematically quantified and attributed uncertainties of SCF DA. They concluded that (i) atmospheric-forcing uncertainty is the largest among the various uncertainty sources examined; (ii) the uncertainty of the model structure as represented by different SCF parameterizations is the second largest; (iii) the DA technique has relatively the least impact on the snow simulations and the Ensemble Adjustment Kalman Filter outperforms the traditional Ensemble Kalman Filter.

To quantify how multisensor LDA improves subseasonal to seasonal (S2S) climate prediction, a series of ensemble-based land–atmosphere-modeling expe-riments were run with the NCAR Community Earth System Model Community Atmospheric Model Version 5 (CAM5)/CLM4 in which land-state variables (such as snow and/or soil moisture) were initialized using 8-year offline DA products. Lin *et al*. [4] conducted three ensemble experiments for seasonal-temperature hindcasts (OL, MOD and GRAMOD). OL did not use snow DA products, MOD used the MODIS DA product, while GRAMOD used the joint MODIS SCF and GRACE TWS DA product. Results show that, in both MOD and GRAMOD, CAM5/CLM4 improves the seasonal-temperature forecasts by 5%–20% in the TP. The TP and its surrounding river basins, high latitudes (e.g. Siberia) and south Asian monsoon areas benefit most from snow DA.

The TP plays an important role in global land–air–sea interactions. Explicitly accounting for such interactions necessitates coupled DA (CDA) systems. Since the Coupled Model Intercomparison Project Phase 5 (CMIP5), large efforts have been devoted to developing and using CDA for decadal climate predictions (DCPs) [5,6]. Based on these efforts, He *et al*. [7] developed a modularized CDA system (Fig. 1c) by applying the dimension-reduced projection (DRP) 4D variational (4DVar) approach [8] to Grid-point Version 2 of the Flexible Global Ocean–Atmosphere–Land System (FGOALS-g2) [9]. Different from other CDA systems, this CDA system used a more advanced adjoint-free 4DVar approach and is capable of assimilating all components except the cryosphere currently.

As an example, a preliminary effort was made to conduct a 62-year CDA cycle for initializing the ocean component of FGOALS-g2, which assimilated the monthly mean analyses of ocean temperature and salinity up to 1000 m deep during 1945–2006 [10]. The CDA improves the spatial distribution and temporal variability of TP summer precipitation significantly. To investigate the contribution of the CDA to the DCP skill, 36 sets of 10-year-long hindcasts were conducted, starting from 1961 and then every year until 1996 with 10 ensemble members. The hindcasts show significant improvements in both precipitation and surface air temperature (SAT) in summer over the TP (Fig. 2e and f). This improvement is mainly due to better initial states of the Atlantic Multi-decadal Oscillation from the CDA, which influence the TP summer precipitation and SAT through large-scale atmospheric circulations.

Despite the initial success of the above prototype LDA and DRP-4DVar-based CDA systems, four main limitations or potential areas for improvement are noted and three grand challenges are pointed out. First, the LDA spatial resolution is too coarse to resolve complex terrains in the TP. Higher resolution is recommended for extending this work. Second, the LDA time span is too short, which is unable to cover observations from new satellites such as the Soil Moisture Active Passive (SMAP), GRACE Follow-on and Fengyun as well as from the recent scientific experiments and field campaigns. Third, the latest advancements in land parameterizations need to be incorporated. Fourth, the CDA currently employs a weak coupling, which should be upgraded to a strongly coupled DA system. While addressing the above limitations, three grand challenges must be kept in mind, namely (i) to use the LDA to guide the development of TP-specific land-surface parameterizations, (ii) to synchronously assimilate observations of atmosphere, ocean, land surface and sea ice while maintaining the stability of long-term coupled integrations especially caused by the high sensitivity of coupled integrations to initial states of the cryosphere and (iii) to apply DA for better understanding and predicting extreme weather and climate events.
